# Effectiveness of video-based teaching in surgery residents: A randomized control trial

**DOI:** 10.12669/pjms.40.10.7856

**Published:** 2024-11

**Authors:** Khurram Niaz, Usman Mahboob, Darakshan Masood, Ali Maqbool

**Affiliations:** 1 Dr. Khurram Niaz FCPS (Surgery), MHPE (KMU) Sheikh Zayed Medical College, Rahim Yar Khan, Pakistan; 2 Dr. Usman Mehboob, Doctorate in Health Professions Education, Institute of Health Professions Education and Research, Khyber Medical University, Peshawar, Pakistan; 3 Dr. Darakshan Masood, FCPS (Gyne), CHPE Bahawal Victoria Hospital, Bahawalpur, Pakistan; 4 Dr. Ali Maqbool BDS, MHPE. Bhitai Dental & Medical College, Mirpurkhas, Pakistan.

**Keywords:** Residents, Surgery, Teaching, Global rating scoring system, Video-based teaching, Recorded video evaluations

## Abstract

**Objective:**

To determine the effectiveness of video-based teaching in surgery residents by comparing it to the routine operating room traditional teaching.

**Methodology:**

The randomized control trial was done at four institutions from September 2022 to March 2023. Interventional group underwent video-based instruction of basic surgical skills whereas the control group was taught through traditional operating room teaching. Pre and post-test mean scores were analyzed on SPSS version 20 through paired t-test. Learning gain was calculated. Supervisors’ perceptions were recorded on survey form regarding direct observations and recorded video evaluations of resident skills on post-test.

**Results:**

Out of sixty (n=60), fifty-five newly inducted surgical residents completed the study including both females (n=13) and male residents (n=42). Video intervention Group (27.93±3.72) and control group (23.07±4.62) both showed improvements in their post-test scores as compared to pretest scores of 13.68±3.25 and 13.52±3.60 respectively. Mean score difference improvement was more in video intervention group (13.9±3.8) in comparison to control group (9.5±4.3) provided both groups exhibits improvements in all seven domains of the global rating system (reflected by the p <0.0001). However, learning gain of 65% was observed in intervention group as compared to learning gain (41%) of control group. Evaluators(n=5) observed that recorded video evaluations helped to provide integrated feedback, despite being time (40%) and resource intensive (60%).

**Conclusion:**

Video-based teaching has higher learning gain irrespective of the fact that both groups exhibit statistically significant results in all seven domains of the global rating system. Recorded video evaluation was found feasible and reliable tool for formative assessment.

## INTRODUCTION

The Covid pandemic has changed the social & educational fabric of medical teaching institutions since its outbreak in 2019. Surgical education of newly inducted residents is affected in the form of limited personal interaction, reduced OR timings, delayed elective admissions, shorter operating lists, vaccination and isolation protocols, etc.^1^

The conventional surgical training of apprenticeship and the ‘Halstedian’ model (sees one, do one & teach one) struggle with providing well-organized and uniform training to all trainees.^2^ In developed countries, the financial cost of training directly in operation theatres and the fixed 48-work hours/week by accredited bodies have pushed educators for parallel strategies to augment teaching. Simulation-based and computer-assisted learning is promoted to acquire the desired competency in tension-free environment.^3^,^4^ Literature mentioned the self-directed video learning of surgical skills as a sole learning strategy in small cohorts, but heterogeneity was found in terms of its feasibility and implementation.^5^,^6^

Video recordings are in use for teaching in laparoscopic and arthroscopic surgeries, however, their use in trainee performance evaluation has not been explored for its effectiveness.^7^ Recorded video evaluations can be convenient for reviewing trainees’ performance at a later stage at remote places but can put additional time demands on faculty.^8^,^9^

Due to overburdened public sector hospitals with limited faculty, face-to-face interaction for teaching and evaluating surgical skills to newly inducted trainees seems less feasible.^10^ Despite beating the traditional methods of surgical skills teaching and assessment an innovative idea of video-based instruction and evaluation is presented here for teaching the technical skills of surgery to the newly inducted residents of surgery. Therefore, we determined the effectiveness of video-based teaching in surgery residents by comparing it to the routine operating room traditional teaching.

## METHODS

This randomized control trial was conducted in the surgical departments of four tertiary care hospitals including Nishtar Hospital (Multan), Sheikh Zayed Hospital (Rahim Yar Khan), Bahawal Victoria Hospital (Bahawalpur) and Combined Military Hospital (Bahawalpur) These hospitals cater large number of emergency surgical cases in the south Punjab region.

### Ethical approval:

All residents and supervisors were informed about the study design, privacy issues, goals, and expectations. Participants participated in the study after signing a written consent. The patients were also informed that junior residents are doing some basic steps of surgery under a senior supervisor.

### Ethical Approval:

We got approval from the Ethics Committee of the Khyber Medical University (DIR/KMU-AS&RB/EV/001712 dated 1^st^ July, 2022) and the pertinent medical colleges (QAMC/2049 dated 2^nd^ Feb, 2023 - NMU/2549 dated 25^th^ February, 2023 - SZMC/622/IRB/SZH dated January 20^th^ 2023).

### Trial Registration:

As this was a trial for the assessment of skills and knowledge of the residents, we didn’t register it online as per guidelines of the International Committee of Medical Journal Editors (ICMJE), (Available at: https://www.icmje.org/about-icmje/faqs/clinical-trials-registration/)

### Participants:

### Trainees:

The newly inducted general surgical residents (6-8 Months) of FCPS and MS Programme of the College of Physicians and Surgeons Pakistan (CPSP) and the University of Health Sciences Punjab (UHS) participated in the study.

### Assessors:

Supervisors of the College of Physicians and Surgeons & University of Health Sciences, Punjab were included in the study for direct and recorded video evaluation of surgical skills on a global rating system. Global Rating System (GRS) is a Likert scoring system that was used to assess the seven learning domains of technical skills of surgery namely: respect for tissue, time and motion, instrument handling, knowledge of instruments, flow of operation, use of assistants, and knowledge of specific procedure.

### Sample Size:

We used the WHO “sample size determination in health studies” with a 95% confidence interval and 80% power of Study. Sixty surgical residents (30 Residents in each group “A”&”B” were enrolled in the study with a 20% attrition rate. Five out of sixty residents did not follow the process so were excluded.

Two groups were formulated by random sampling (lottery method) of residents fulfilling the inclusion criteria of six months of house jobs in surgery and induction through central policy. Group “A” the intervention group was taught through the videos of basic surgical skills (7-14 days). The videos shared with the intervention group were on Instrument handling, Incision handling, Simple interrupted suturing and vertical mattress suturing While Group “B” continued their training through routine traditional teaching of skills in operation theatre.

### Data Collection:

**Fig.1:**
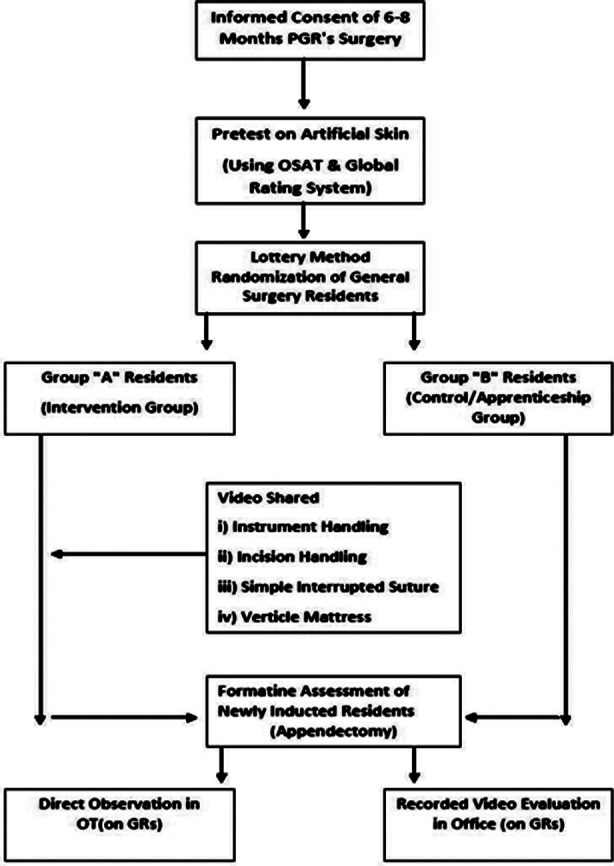
Flowchart for Study Design Results.

The Objective Structured Assessment of Technical Skills (OSATS) was used for objective skill assessments. Each of the seven domains of the global rating scoring system was scored on a 5-point scale with a total of 35 points for novice residents. All residents underwent a pretest and posttest on artificial skin and got scores on the global rating scoring system (Fig.1). The responses of supervisors were recorded on a predesigned proforma for recorded video evaluation. Their responses were recorded in the form of yes and No on their experience of various aspects of recorded video evaluation of the basic surgical skills of residents.

### Data Analysis:

We used the SPSS version 20 for data analysis and a paired t-test was applied for quantitative statistical analysis of interventional and control/Apprenticeship groups. The evaluator’s perceptions were recorded in the form of yes or no, on predesigned proformas and presented as frequency tables.

### Pilot Testing:

Pilot testing of the study was done with Eighteen (18) residents of surgical unit II of Sheikh Zayed Medical College and Hospital Rahim Yar Khan. Five supervisors of the College of Physicians and Surgeons Pakistan (CPSP) and the University of Health Sciences (UHS) were involved in the pretest and post-test evaluation directly and on recorded videos of basic surgical skills. For recorded videos, one resident with an iPhone was deputed to make video clips of skills, and later video cutter was used to merge the different steps of performed skills. The Likert scale of the global scoring system (GRS) was used for resident scoring.

## RESULTS

Out of 55 residents, 42 (76.36%) were males and 13 were females (23.63%). Out of these eight residents had undergone more than three months of training, (Table-I). The means score of the two groups is shown in Table-II based on gender. The mean score improvement for both genders has been observed. The perception of residents for the video based equipment shows positive views toward it, (Table-III).

**Table-I:** Frequency and Percentage of Demographic data of study subjects.

Institution	Duration of training			Gender		Total n=55	Frequency (%)

	< 1 month	1-3 Month	>3Month	Male	Female		
Hospital A MTN	10	----	----	09	01	n=10	18%
Hospital B BWP	10	04	02	13	03	n=16	29%
Hospital C RYK	11	10	06	19	08	n=27	50%
Hospital D BWP	01	01	---	01	01	n=2	4%

**Table-II:** Gender base Mean scores for Video intervention and control group.

Institution	Gender	Group-A			Group-B	

		Pre test	Post test	Gender	Pre test	Post test
NMU, Multan	M (n=5)	14.6	2.8	M(n=4)	15	27.2
	F (n=0)	---	---	F (n=1)	16	28
SZMC, R. Y. Khan	M (n=11)	12.09	24.1	M(n=8)	13.22	31.11
	F (n=3)	12.66	27.66	F(n=5)	13.25	21
BVH, Bahawalpur	M (n=7)	14.57	29.71	M(n=6)	613	22
	F (n=1)	19	29.00	F (n=2)	12.5	24
CMH, Bahawalpur	M (n=0)	----	----	M(n=1)	12	18
	F (n=1)	16	34	F (n=0)	----	----

**Table-III:** Perception of evaluators on recorded video evaluations.

Evaluators Perceptions	Disagree (%)	Equivocal (%)	Agree (%)
Recorded video evaluation is more time-consuming	60	20	20
Resource intensive	20	-*	80
Observation Bias	20	-	80
Comparable experience for all students	-	40	60
Realistic workload for assessors	20	20	60
Can be used as a formative assessment	20	-	80
Can be used as Summative Assessment	40	20	40
Reliable	20	20	60
Feasible	40	-	60
Generate authentic experience	20	40	40
Provides integrated Feedback	-	20	80
Addresses the patient safety issues	-	40	60
Helpful in portfolio maintenance	-	-	100

* The – sign means that none of the participants filled these cells.

## DISCUSSION

Traditional surgical training programs tend to provide skill development alongside healthcare provision.^11^,^12^ Keeping this in mind we focused on investigating a practical e-learning method suiting the resident’s tough schedule focusing on the basic surgical technique of handling incisions, instruments and sutures, voiced over by an expert to promote flexible learning. Our study has examined the largest number of study subjects in this domain which is fifty-five with good learning gain.

In our study, out of 55 residents who participated, the majority were males (n=42) in four institutions. The mean score improvement was significant for female residents (n=13) in two institutions in the video intervention group. While in the control group, male residents showed significant improvement from the pre-test to the post-test mean. The rest of the results endorsed the already published data that learning is not affected by gender and it’s not a confounding factor.^13^ Most of the residents who participated in our study were novices (<one month) and their mean post-test scores improved significantly in both groups as compared to those who were more exposed to the clinical ward system (one month –three-month duration).^14^

Both the video interventional and control groups showed improvement in post-test scores. Despite a higher post-test score and good learning gain for the video intervention group, there was no statistically significant difference between those who had access to the expert - led videos (p<0.0001) and those who trained through the apprenticeship model as the control group (p<0.0001). Similar findings were found in a study on a small number of study subjects.^9^ As both groups showed improvement in their mean post-test scores, it can be inferred that simply applying the basic surgical skills on simplex procedures ten days apart was enough to improve the skills of residents. The chances of video sharing between interventional and control group residents were unlikely but we cannot rule it out with certainty.


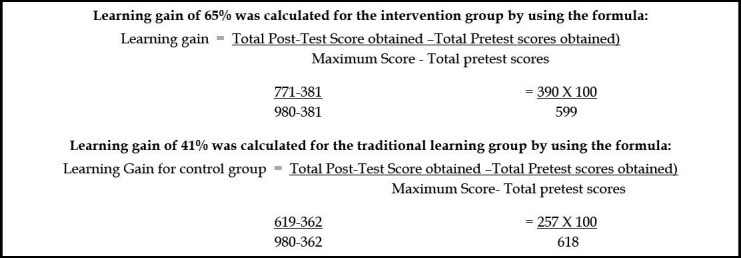


Training directly in operation theatres and performing technical skills after getting acquainted outside is advocated nowadays.^15^ Online teaching has attracted educationists in the pandemic era due to restrictions on face- to-face interaction.^16^ There is limited evidence regarding its practicality and usefulness.^17^ Studies have assessed its use as a sole method or in conjunction with traditional learning techniques of novice surgeons.^18^.^6^,^19^ Few studies mentioned the contrary results with fewer study subjects.^20^,^21^

The video intervention group showed statistically significant improvement in all domains of the global rating scoring system between pre-test and post-test scores but mostly it was in domains of respect for tissue, instrument knowledge, and its handling compared to the control group. This contradicts to most of the available data where studies showed statistically insignificant improvement in post-test scores.^22^,^23^

The direct observation of trainees, in the operating room is the Gold Standard, has many challenges like single rater limitation and patient safety issues besides the examiner and examinee burnout. In search of an effective evaluation tool for surgical skills, we compared the real-time recorded video evaluation and direct observation in a post-test on the same study subject. The evaluator responses from different institutions showed that most of them considered recorded video evaluation as a feasible tool for skill assessment and integrated feedback. Surprisingly there is no consensus on one standardized evaluation method to check the effectiveness of skills training. Pre and post-test approach is not popular as it requires a lot of time &resources.^24^ This method has been shown less effective by few studies in comparison to procedure-based assessments and direct observed procedural skills.^25^ In operation theatres, the supervisor can look for patient safety issues and evaluate recorded videos later.^26^,^27^

Despite similar results in both groups, in our study the improved post test scores and learning gains of video-based instruction mandates further exploration for surgical curriculum development. With patient consent, we can assess the recorded video evaluations against directly observed procedural skills in complex surgeries like cholecystectomy, etc. and integrate it in portfolios. Recorded video evaluations have great potential to be established as an assessment tool in resource intensive open surgery settings. It can be readily extended to laparoscopic and robotic surgery for the formative assessment of surgery residents. Their role can be further explored for minimally invasive surgery training evaluations without observation bias.

### Limitations:

The limitation of our study is that it is conducted at multiple centers with different evaluators without blinding. Interrater reliability can be varied despite using the benchmark that all evaluators were accredited supervisors from the College of Physicians and Surgeons (CPSP) Pakistan & University of Health Sciences Lahore. Lastly, the senior evaluators preferred the recorded video evaluation instead of direct observation in operation theatres which can impact the results due to the Hawthorne effect i.e., the residents being observed modified their behavior psychologically when taught through innovative video based teaching’

## CONCLUSION

In our study video based teaching has higher learning gain irrespective of the fact that both groups exhibits statistically significant results in all seven domains of the global rating system. Good learning gain can endorse this potential pedagogic method of self-directed learning in future. Most Evaluators considered recorded video evaluation a feasible and reliable tool for formative assessment and integrated feedback despite being resource and time intensive.

### Authors Contribution:

**UM, KN** conceived, designed, and did statistical analysis & editing of manuscript, is responsible for integrity of research.

**KN, DSM, AM**, did data collection and wrote multiple drafts of the manuscript.

**UM** edited the drafts.

All authors reviewed the final draft of the manuscript, approved for publication and are responsible for the integrity of the study.
